# Clinical and virological impact of single and dual infections with influenza A (H1N1) and SARS-CoV-2 in adult inpatients

**DOI:** 10.1371/journal.pntd.0009997

**Published:** 2021-11-29

**Authors:** Jiazhen Zheng, Fengjuan Chen, Keyi Wu, Jiancheng Wang, Furong Li, Shan Huang, Jianyun Lu, Jinghan Huang, Huamin Liu, Rui Zhou, Zhiwei Huang, Bingyao Meng, Zelin Yuan, Xianbo Wu

**Affiliations:** 1 Department of Epidemiology, School of Public Health (Guangdong Provincial Key Laboratory of Tropical Disease Research), Southern Medical University, Guangzhou, Guangdong, China; 2 School of Public Health (Shenzhen), Sun Yat-sen University, Shenzhen, China; 3 Department of Medical Administration, Guangzhou Eighth People’s Hospital, Guangzhou, Guangdong, China; 4 Department of Intensive Care Unit, The Third Affiliated Hospital of Southern Medical University, Guangzhou, Guangdong, China; 5 School of Medicine, Southern University of Science and Technology, Shenzhen, China; 6 P3 biosafety laboratory, School of Public Health (Guangdong Provincial Key Laboratory of Tropical Disease Research), Southern Medical University, Guangzhou, Guangdong, China; 7 Department of Infectious Disease Control and Prevention, Guangzhou Center for Disease Control and Prevention, Guangzhou, Guangdong, China; 8 Department of Biostatistics, School of Public Health, Boston University, Boston, United States of America; 9 Department of Toxicology, School of Public Health (Guangdong Provincial Key Laboratory of Tropical Disease Research), Southern Medical University, Guangzhou, Guangdong, China; The University of Hong Kong, CHINA

## Abstract

Severe acute respiratory syndrome coronavirus 2 (SARS-CoV-2) mimics the influenza A (H1N1) virus in terms of clinical presentation, transmission mechanism, and seasonal coincidence. Comprehensive data for the clinical severity of adult patients co-infected by both H1N1 and SARS-CoV-2, and, particularly, the relationship with PCR cycle threshold (Ct) values are not yet available. All participants in this study were tested for H1N1 and SARS-CoV-2 simultaneously at admission. Demographic, clinical, treatment, and laboratory data were extracted from electronic medical records and compared among adults hospitalized for H1N1 infection, SARS-CoV-2 infection and co-infection with both viruses. Ct values for viral RNA detection were further compared within SARS-CoV-2 and co-infection groups. Score on seven-category ordinal scale of clinical status at day 7 and day 14 were assessed. Among patients with monoinfection, H1N1 infection had higher frequency of onset symptoms but lower incidence of adverse events during hospitalization than SAR-CoV-2 infection (P < 0.05). Co-infection had an increased odds of acute kidney injury, acute heart failure, secondary bacterial infections, multilobar infiltrates and admittance to ICU than monoinfection. Score on seven-category scale at day 7 and day 14 was higher in patients with coinfection than patients with SAR-CoV-2 monoinfection (P<0.05). Co-infected patients had lower initial Ct values (referring to higher viral load) (median 32) than patients with SAR-CoV-2 monoinfection (median 36). Among co-infected patients, low Ct values were significantly and positively correlated with acute kidney injury and ARDS (P = 0.03 and 0.02, respectively). Co-infection by SARS-CoV-2 and H1N1 caused more severe disease than monoinfection by either virus in adult inpatients. Early Ct value could provide clues for the later trajectory of the co-infection. Multiplex molecular diagnostics for both viruses and early assessment of SAR-CoV-2 Ct values are recommended to achieve optimal treatment for improved clinical outcome.

## Author summary

The baseline enrolled 505 patients admitted to Guangzhou Eighth People’s Hospital (Guangzhou, Guangdong) with a diagnosis of COVID-19 or H1N1. All the patients were tested by both viruses at admission. Demographic, clinical, treatment, and laboratory data were extracted from electronic medical records and compared among adults (≥18 years) hospitalized for H1N1 infection (*n* = 220), SARS-CoV-2 infection (*n* = 249) and co-infection with both viruses (*n* = 36). The prevalence rate of H1N1 co-infection was 12.6% (36/285) among patients hospitalized with COVID-19. Co-infection affected a predominantly older age group and was associated with poorer clinical outcome. We also described the viral load trajectory in patients with diverse types of infection. Lower initial Ct values (higher viral loads in nasopharyngeal swabs) of co-infected patients was found to be associated with a higher number of adverse events and clinical symptoms. Considering the COVID-19 pandemic and a simultaneous epidemic of seasonal influenza, the data in China may critically inform future therapeutic or prophylactic strategies, especially for other developing countries.

## Introduction

Influenza is a contagious respiratory disease that is widespread worldwide. Despite the advances in medical technology, influenza causes considerable hospitalizations and mortality [1,2]. H1N1 is a subtype of influenza A virus, causing respiratory infections and has caused two pandemics over the past 102 years [3,4]. The most recent pandemic of H1N1 influenza occurred in 2009 and affected 60.8 million people, resulting in 284,000 deaths worldwide [5,6]. The Northern Hemisphere faces the prospect of the COVID-19 pandemic and a simultaneous epidemic of seasonal influenza [7,8], and the management of the disease was complicated by the diversity in “influenza-like” clinical manifestations [9], resulting in enormous challenge in preventing and controlling the influenza epidemic [10].

Both SARS-CoV-2 and influenza viruses are respiratory tract viruses that invade the host through specific receptors, causing pneumonia in severe cases. Furthermore, the pathogenesis and receptors of these two viruses responsible for causing pneumonia are different; the SARS-CoV-2 infection affects the lower respiratory tract while the influenza infection primarily attacks the upper respiratory tract. There is no receptor competition, and viral co-infection occurs without any difficulty [11].

In Northeastern Iran, Hashemi et al evaluated the presence of influenza A virus in 105 dead SARS-CoV-2 positive patients, in which 23 patients (22.3%) were coinfected with the influenza A virus identified, implicated a potential high prevalence of coinfection with influenza A virus. A study on forty-eight COVID-19 patients, conducted by Alosaimi et al., showed that the prevalence rate of co-infection with H1N1 and SAR-CoV-2 was 71% [12]. However, the prevalence rate may be inaccurate because of the limited sample size. Despite a move towards considering the evidence base for co-infection in the past year, most studies were case series [9,13–16], and a knowledge gap exists in the clinical outcomes of co-infection with H1N1 and SARS-CoV-2 in adult patients. Further, we currently lack a clear understanding of the relationship between SARS-CoV-2 PCR cycle threshold (Ct) values and clinical severity in patients co-infected by SARS-CoV-2 and H1N1.

In previous reports, we described the PCR Ct values dynamics among COVID-19 patients by clinical conditions [17,18]. In the current study, we conducted a retrospective cohort study on COVID-19 (original SARS-CoV-2 but not variants) and H1N1 inpatients with the purpose of answering the following three questions: What is the prevalence rate of H1N1 co-infection in COVID-19 patients? What are the association between different infection types and clinical severity and outcomes in H1N1 and COVID-19 patients? What do Ct values tell us about the clinical severity of co-infection by COVID-19 and H1N1? The answers to these questions are essential for formulating the principles of optimize therapy for adult patients with COVID-19 and H1N1 infections.

## Methods

### Ethics statement

The study protocol was reviewed and approved by Ethics Committees of Guangzhou Eighth People’s Hospital (No. 202002137). Signed informed consent of participants or their guardians was waived because of the nature of retrospective study.

### Study population

This retrospective study analyzed inpatients admitted to the Guangzhou Eight People’s Hospital with main diagnosis of H1N1 or COVID-19 infection. All patients were tested for H1N1 and SARS-CoV2 virus at admission. After excluding patients who were minors (≤18 years), with missing clinical data or immune deficiency, 220 H1N1-infected patients between January 2020 and November 2020 were enrolled in this study. For patients with COVID-19, we excluded patients who were minors (≤18 years), with missing clinical data or immune deficiency, and then 285 COVID-19 patients admitted to the hospital between January 2020 and April 2020 were enrolled. A total of 36 patients co-infected with H1N1 and COVID-19 (belong to the group of 285 COVID-19 patients) were used as the comparator group.

### Clinical data collection and definitions

Patients infected by SARS-CoV-2 were assessed by trained nurses using diary cards that captured data on a seven-category ordinal scale at day 7 and day 14. Ordinal scales have been used in clinical trials in patients hospitalized with severe influenza and COVID-19 [19,20]. The seven-category ordinal scale consisted of the following categories: one point, not hospitalized with resumption of normal activities; two points, not hospitalized, but unable to resume normal activities; three points, hospitalized, not requiring supplemental oxygen; four points, hospitalized, requiring supplemental oxygen; five points, hospitalized, requiring nasal high-flow oxygen therapy, noninvasive mechanical ventilation, or both; six points, hospitalized, requiring ECMO, invasive mechanical ventilation, or both; and seven points, death. We used the WHO-ISARIC (World Health Organization–International Severe Acute Respiratory and Emerging Infections Consortium) case record form (https://isaric.tghn.org) to record other clinical data [21]. We reviewed electronic and written medical records for all subjects. The electronic medical records were reviewed by a trained team of physicians. Data collected included demographic details, comorbid illnesses, presenting symptoms and signs, blood biochemical indexes, antiviral and antibiotic use, vasoactive drugs received (dobutamine or noradrenaline), intensive care unit (ICU) admission, hospital length of stay, requirement for ventilatory support, and occurrence of adverse events. Pneumonia was defined as the presence of pulmonary infiltrates on chest imaging. Acute respiratory distress syndrome (ARDS) was defined based on the Berlin definition [22]. Immune deficiency was defined as organ transplant, active therapy for malignancy, and high-dose steroids/other iatrogenic drugs. Prolonged hospitalization was defined as the length of stay in the hospital longer than 20 days. Co-infection with H1N1 and COVID-19 is defined as positive nucleic acid detection for both viruses at admission. Other adverse events were classified according to the National Cancer Institute Common Terminology Criteria for Adverse Events, version 4.0.

### Testing process and analysis

H1N1 virus infection was confirmed by the analysis of nasopharyngeal swabs, sputum, bronchoalveolar lavage fluid samples using Influenza A Virus Nucleic Acid Detection kit (Liferiver) [23] at admission. Patients’ nasopharyngeal swab specimens were collected by CDC clinicians for SARS-CoV-2 nucleic acid detection by RT-PCR at admission and once every two or three days during hospitalization. The detailed protocol of the RT-PCR is described in S1 Appendix and other study [24]. The threshold refers to the critical values of fluorescence signal in exponential growth period. Ct values refers to the number of cycles when the fluorescence signal reaches the threshold. A Ct-value less than 37 was defined as positive, Ct-values ≥40 was defined as negative, and a medium load (Ct-values 37–40) was an indication for retesting [25]. Lower Ct values refers to higher viral load.

### Statistical analysis

Continuous variables are described as medians (interquartile ranges, IQRs). Categorical variables are presented as frequencies and percentages. Comparisons of proportions were performed with chi-square and Fisher’s exact tests; continuous variables were compared using the Mann–Whitney U test. All probabilities were two-tailed, with statistical significance defined as P ≤ 0.05. Binary logistic regression was performed to estimate the odds ratio (OR) and 95% confidence interval (CI) for comparison of clinical hospitalization outcomes in H1N1-infected, SARS-CoV-2 infected and co-infected groups. Adjustment factors included age, sex, comorbidities and smoking history. These risk factors were previously shown to be associated with the clinical prognosis of influenza and COVID-19 patients and served as confounders [26,27]. Because Ct values are semi-quantitative, differences in Ct values between groups were assessed nonparametrically using the Mann-Whitney U test. Correlations between Ct values and age were assessed nonparametrically using tie-corrected Spearman rank correlation coefficients. All analyses were performed using STATA, version 14.

## Results

### Comparison between patients infected with H1N1 and SARS-CoV-2

Demographic characteristics and comorbidities prior to admission of all hospitalized patients are presented in Table 1. The median ages of H1N1 and SARS-CoV-2-infected patients were 50.0 years (IQR, 30.0–65.0, range 20.0–90.0) and 47.0 years (33.0–61.0, range 18.0–90.0). The duration from disease onset to admission was longer in patients infected with H1N1 than those infected with SARS-CoV-2 (4.0 days vs. 3.0 days). At admission, H1N1-infected participants showed symptoms more frequently than SARS-CoV-2-infected participants, except for dry cough and diarrhea. In addition, patients with H1N1 had higher temperature at admission than patients infected with SARS-CoV-2 (median 37.8°C [36.8–38.2] vs. 37.2°C [36.8–37.6]). For infection-related biomarkers, H1N1 patients’ C-reactive protein and procalcitonin levels (median C-reactive protein level, 44.96 mg/L [20.36–77.75]; median procalcitonin level, 0.16 ng/mL [0.05–0.61]) were higher than patients infected with COVID-19 (median C-reactive protein level, 32.08 mg/L [12.08–36.30]; median procalcitonin level 0.07 ng/mL [0.04–24.7], Table 2). SARS-CoV-2-infected patients more frequently received antiviral therapy (84.74% vs. 76.82%, P = 0.029, Table 3) and oxygen therapy (62.65% vs. 40.91, P < 0.001). In multivariable analysis, the odds of multilobar infiltrates were higher in SARS-CoV-2-infection than H1N1 infection (Table 4).

**Table 1:** Demographic data and pre-existing conditions of hospitalized patients with H1N1, SARS-CoV-2 and co-infection.

Variable	H1N1 infection (n = 220)	SARS-CoV-2 infection (n = 249)	P-value H1N1 vs SAR-CoV-2	Co-infection (n = 36)	P-value SARS-CoV-2 vs co-infection	P-value H1N1 vs co-infection
**Age (years, median, IQR)**	50.0 (30.0–65.0)	47.0 (33.0–61.0)	0.4968	56.0 (39.5–66.0)	**0.0265**	0.1349
18–30 y (n, %)	40 (18.2)	42 (16.9)		1 (2.8)		
31–40 y (n, %)	37 (16.8)	48 (19.3)		8 (22.2)		
41–50 y (n, %)	33 (15.0)	49 (19.7)		6 (16.7)		
51–60 y (n, %)	39 (17.7)	44 (17.7)		6 (16.7)		
>60 y (n, %)	71 (32.3)	66 (26.5)		15 (41.7)		
**Male (n, %)**	129 (58.64)	139 (55.82)	0.539	19 (52.78)	0.731	0.509
**Comorbidities (n, %)**						
Hypertension	40 (18.18)	40 (16.06)	0.543	9 (25.00)	0.184	0.335
Cerebrovascular disease	20 (9.09)	11 (4.42)	**0.042**	2 (5.56)	0.76	0.483
Diabetes Mellitus	22 (10.00)	13 (5.22)	**0.049**	5 (13.89)	**0.046**	0.481
Coronary heart disease	8 (3.64)	9 (3.61)	0.99	4 (11.11)	**0.044**	**0.049**
Hyperlipidemia	18 (8.18)	9 (3.61)	**0.034**	3 (8.33)	0.188	0.975
COPD	19 (8.64)	8 (3.21)	**0.012**	5 (13.89)	**0.004**	**0.316**
Chronic Liver disease	26 (11.82)	17 (6.83)	0.062	8 (22.22)	**0.002**	0.088
Chronic kidney disease	14 (6.36)	8 (3.21)	0.107	3 (8.33)	0.136	0.660
**Smoking history**	38 (17.27)	27 (10.84)	**0.044**	4 (11.11)	0.962	0.355
**Days from disease onset to admission (median, IQR)**	4.0 (2.0–7.0)	3.0 (1.0–6.5)	**0.0012**	3.0 (2.0–7.0)	0.453	0.363

COPD denotes chronic obstructive pulmonary diseases, IQR interquartile range.

**Table 2:** Signs and symptoms and laboratory findings in admission of patients with H1N1, SARS-CoV-2 and co-infection with both viruses.

Variable	H1N1 infection (n = 220)	SARS-CoV-2 infection (n = 249)	P-value H1N1 vs SAR-CoV-2	Co-infection (n = 36)	P-value SARS-CoV-2 vs co-infection	P-value H1N1 vs co-infection
**Signs and Symptoms (n, %)**						
Fever	182 (82.73)	121 (48.59)	**<0.001**	23 (63.89)	0.086	**0.009**
Median temperature (IQR)—°C	37.80 (36.80–38.15)	37.20 (36.80–37.60)	**<0.001**	37.65 (36.95–38.05)	**0.038**	0.997
Fatigue	105 (47.73)	66 (26.51)	**<0.001**	12 (33.33)	0.390	0.108
Dry cough	123 (55.91)	134 (53.82)	0.649	25 (69.44)	0.078	0.127
Expectoration	99 (45.00)	53 (21.29)	**<0.001**	10 (27.78)	0.380	0.053
Chills	86 (39.09)	59 (23.69)	**<0.001**	13 (36.11)	0.109	0.734
Myalgia	88 (40.00)	25 (10.04)	**<0.001**	9 (25.00)	**0.010**	0.085
Headache	77 (35.00)	37 (14.86)	**<0.001**	5 (13.89)	0.878	**0.012**
Sore throat	66 (30.00)	41 (16.47)	**<0.001**	5 (13.89)	0.694	**0.045**
Diarrhea	2 (0.91)	10 (4.02)	**0.033**	1 (2.78)	0.718	0.334
Respiratory rates (beats/min, median, IQR)	20.0 (19.0–22.0)	20.0 (18.0–20.0)	**<0.001**	20.0 (18.0–20.0)	0.267	0.108
**Laboratory finding (median, IQR)**						
PO_2_ (mmHg)	90.5 (82.0–110.0)	92.5 (80.4–107.0)	0.8637	86.8 (72.5–104.4)	0.221	0.234
HB (g/L)	135.0 (122.0–143.0)	136.0 (123.5–146.0)	0.3081	129.0 (118.0–141.0)	**0.041**	0.148
WBC count (× 10^9^ cells/L)	6.18 (4.41–8.52)	4.81 (3.93–6.30)	**<0.001**	5.55 (4.30–6.80)	0.092	0.215
Neutrophil count (× 10^9^ cells/L)	4.05 (2.65–6.39)	2.83 (2.09–3.89)	**<0.001**	3.67 (2.71–5.10)	**0.008**	0.213
Lymphocyte count (× 10^9^ cells/L)	1.01 (0.60–1.59)	1.42 (1.02–1.91)	**<0.001**	1.15 (0.96–1.72)	0.089	0.085
CD45+(μg/L)	1317.0 (869.0–1629.0)	1439.5 (1183.0–1778.0)	**0.021**	1195.0 (1037.0–1840.0)	0.349	0.514
**Infection-related biomarker**						
C-reactive protein level (mg/L)	44.96 (20.36–77.75)	32.08 (12.08–36.30)	**<0.001**	41.2 (29.10–70.20)	**0.035**	0.400
ESR (mm/h)	23.00 (12.00–50.0)	27.2 (15.00–43.10)	0.5007	18.10 (10.00–32.10)	0.118	0.447
Procalcitonin level (ng/mL)	0.16 (0.05–0.61)	0.07 (0.04–24.70)	**0.0332**	0.15 (0.04–57.90)	0.154	0.955

SBP denotes systolic blood pressure, DBP diastolic blood pressure, WBC white blood cell, ESR erythrocyte sedimentation rate

IQR interquartile range.

**Table 3:** Treatment and clinical outcome of patients with Influenza H1N1, SARS-CoV-2 and co-infection with both viruses.

Variable	H1N1 infection (n = 220)	SARS-CoV-2 infection (n = 249)	P-value H1N1 vs SAR-CoV-2	Co-infection (n = 36)	P-value SARS-CoV-2 vs co-infection	P-value H1N1 vs co-infection
**Treatment and clinical course (n, %)**						
Intravenous antibiotics	175 (79.55)	190 (76.31)	0.399	30 (83.33)	0.348	0.598
Vasoactive agents	32 (17.68)	46 (18.47)	0.833	13 (36.11)	**0.015**	**0.013**
Antiviral therapy	169 (76.82)	211 (84.74)	**0.029**	33 (91.67)	0.268	**0.043**
Oxygen therapy	90 (40.91)	156 (62.65)	**<0.001**	29 (80.56)	**0.035**	**0.001**
Ventilation support	6 (2.73)	11 (4.42)	**0.328**	7 (19.44)	**0.001**	**<0.001**
Length of stay in hospital (days, median, IQR)	7.0 (4.0–10.0)	17.5 (13.0–24.0)	**<0.001**	20.0 (16.0–27.5)	**0.02**	**0.001**
**Adverse event (n, %)**						
Multilobar infiltrates	65 (29.55)	126 (50.60)	**<0.001**	24 (66.67)	0.071	**0.001**
Secondary bacterial infections	10 (4.5)	8 (3.21)	0.453	5 (13.89)	**0.004**	**0.027**
Acute kidney injury	5 (2.27)	7 (2.81)	0.712	4 (11.11)	**0.016**	**0.008**
Acute heart failure	3(1.36)	5(2.01)	0.591	3(8.33)	**0.032**	**0.01**
ARDS	5 (2.27)	11 (4.42)	0.202	3 (8.33)	0.31	0.053
Admittance to ICU	9 (4.09)	13 (5.22)	0.564	5 (13.89)	**0.046**	**0.017**
30-day mortality	1 (0.45)	0	0.287	1 (2.78)	**0.008**	0.142

Ventilation support include noninvasive ventilation, invasive ventilation and ECMO. ECMO denotes extracorporeal membrane oxygenation, ICU intensive care unit, IQR interquartile range.

**Table 4:** Risk of clinical adverse events in patients with Influenza H1N1, SARS-CoV-2 and co-infection with both viruses.

	Adjusted odds ratio (95% CI)	P
**Acute kidney injury**		
H1N1 infection	1 (ref)	NA
SARS-CoV-2 infection	3.68 (0.88–15.43)	0.075
Co-infection	9.69 (1.72–54.75)	**0.010**
**Acute heart failure**		
H1N1 infection	1 (ref)	NA
SARS-CoV-2 infection	3.43 (0.63–18.58)	0.154
Co-infection	11.19 (1.62–77.32)	**0.014**
**Secondary bacterial infection**		
H1N1 infection	1 (ref)	NA
SARS-CoV-2 infection	0.83 (0.29–2.28)	0.712
Co-infection	3.60 (1.09–11.91)	**0.036**
**Multilobar infiltrates**		
H1N1 infection	1 (ref)	NA
SARS-CoV-2 infection	2.13 (1.44–3.15)	**<0.001**
Co-infection	4.44 (2.07–9.49)	**<0.001**
**ARDS**		
H1N1 infection	1 (ref)	NA
SARS-CoV-2 infection	3.06 (0.92–10.19)	0.068
Co-infection	3.48 (0.71–17.16)	0.125
**Admittance to ICU**		
H1N1 infection	1 (ref)	NA
SARS-CoV-2 infection	1.60 (0.64–4.05)	0.318
Co-infection	3.46 (1.04–11.62)	**0.044**

Adjustment factors included age, sex, comorbidities and smoking history. ARDS denotes acute respiratory distress syndrome.

### Comparison between co-infection and monoinfection

Co-infected patients were older (median 56.0 years vs 47.0 years and 50 years) and had a higher proportion of comorbidities than patients with monoinfection (Table 1). For comparison of the results of routine blood tests, neutrophil count and C-reactive protein levels (3.67×10^9^ cells/L; 41.2 mg/L) were significantly higher in co-infected patients than patients with infected by SARS-CoV-2 alone (2.83×10^9^ cells/L; 32.08 mg/L) (P<0.05), while hemoglobin level was lower (P<0.01). The laboratory findings were not significantly different between co-infected and H1N1 patients.

High proportion of co-infected patients underwent vasoactive agent therapy, antiviral therapy, oxygen therapy and ventilation support (Table 3). Generally, the incidence rates of clinical adverse events were higher in elder co-infected patients. In comparison with H1N1 and COVID-19 patients, co-infection tended to have increased odds of clinical adverse events (e.g., acute kidney injury, acute heart failure, secondary bacterial infections, multilobar infiltrates, and admittance to ICU; Table 4). Patients with co-infection had significantly higher score on 7-category ordinal scale than patients with SARS-CoV-2 monoinfection at day 7 (4.0 vs. 3.0, p = 0.008) and day 14 (3.0 vs. 3.0, p = 0.020) (Table 5).

**Table 5:** Seven-category ordinal scale of clinical status in patients with SARS-CoV-2 monoinfection and co-infection.

	Monoinfection with SARS-CoV-2	Co-infection with SARS-CoV-2 and H1N1	P
Score on seven-category scale at day 7—no. of patients (%)	3.0 (3.0–4.0)	4.0 (3.0–4.0)	**0.008**
2: Not hospitalized, but unable to resume normal activities	47 (18.9)	6 (16.7)	
3: Hospitalization, not requiring supplemental oxygen	113 (45.4)	9 (25.0)	
4: Hospitalization, requiring supplemental oxygen	79 (31.7)	13 (36.1)	
5: Hospitalization, requiring HFNC or noninvasive mechanical ventilation	8 (3.2)	4 (11.1)	
6: Hospitalization, requiring ECMO, invasive mechanical ventilation, or both	2 (0.8)	3 (8.3)	
7: Death	0	1 (2.8)	
Score on seven-category scale at day 14—no. of patients (%)	3.0 (2.0–4.0)	3.0 (2.0–4.0)	**0.020**
2: Not hospitalized, but unable to resume normal activities	105 (42.2)	11 (30.6)	
3: Hospitalization, not requiring supplemental oxygen	83 (33.3)	9 (25.0)	
4: Hospitalization, requiring supplemental oxygen	53 (21.3)	9 (25.0)	
5: Hospitalization, requiring HFNC or noninvasive mechanical ventilation	7 (2.8)	3 (8.3)	
6: Hospitalization, requiring ECMO, invasive mechanical ventilation, or both	3 (1.2)	3 (8.3)	
7: Death	0	1 (2.8)	

ECMO denotes extracorporeal membrane oxygenation, HFNC high-flow nasal cannula for oxygen therapy.

### Analysis of distribution regulation of CT values

**Fig 1:**
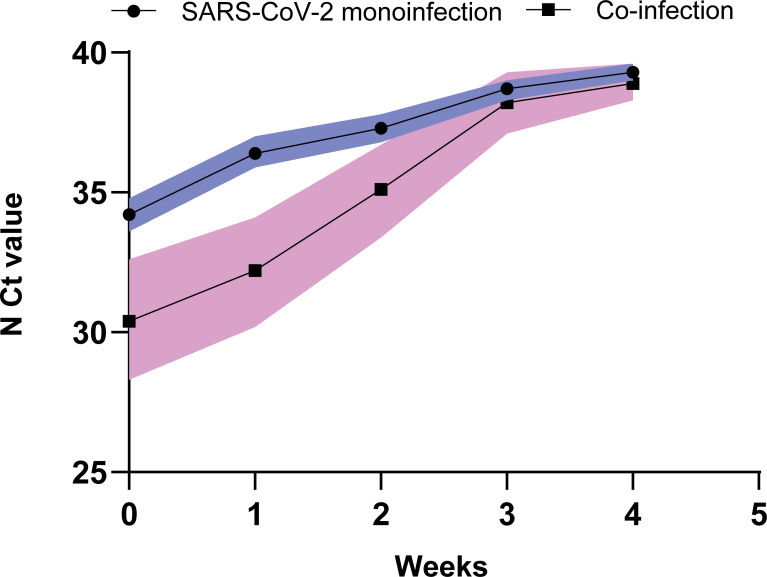
Comparison of viral dynamics between patients with co-infection and SAR-CoV-2 monoinfection. The figure shows temporal changes in median (with 95 CI) Ct value of N gene in different time periods after diagnosis.

**Fig 2:**
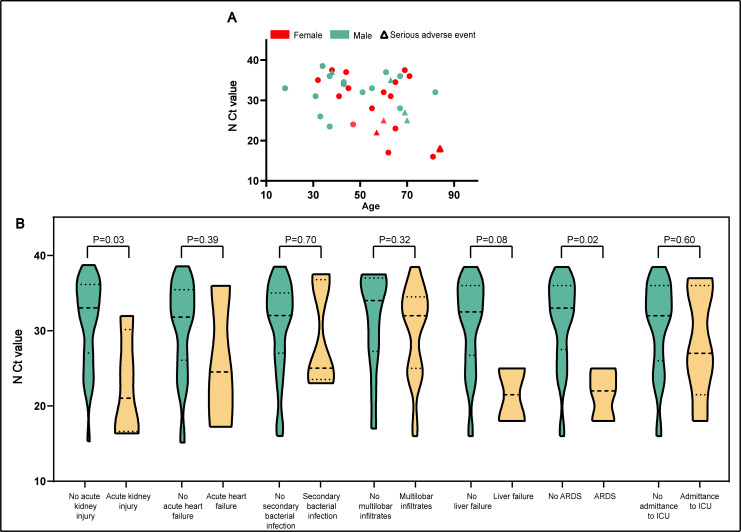
Participants’ Cycle Threshold Values from Nasopharyngeal Swabs, by age, symptoms and adverse events in patients co-infected by COVID-19 and H1N1. Panel A, cycle threshold (Ct) values for SARS-CoV-2 rRT-PCR target probes N are plotted against the age of inpatients. Serious adverse events were marked by a triangle which was defined as the occurrence of liver failure, ARDS or admittance to ICU. Age was not significantly correlated with Ct values (correlation coefficient = -0.0751, P = 0.2065). Panel B, distributions of initial N probe Ct values among co-infected participants suffering from specific adverse event are displayed by violin plot. Dotted lines on upper and lower sides illustrate interquartile ranges, both ends of violin body illustrated 95% distributions, and medians are marked by the dotted line in the middle. Width of violin body referred to number of participants.

Ct values at admission were lower in co-infected patients (median 32 [25.5–35.5]) than patients with SAR-CoV-2 monoinfection (median 36 [32–38]) (Fig 1), indicating higher initial viral load in co-infected patients. In the subsequent monitoring, the Ct value of the two groups have been increasingly similar. Although we observed a slight reverse association between the initial Ct values of the co-infected patients and their age, but this association was not significant (correlation coefficient = -0.0751, P = 0.2065) (Fig 2A). Participants suffered from acute kidney injury and ARDS had significantly lower initial Ct values than participants who did not meet this criterion (median Ct values of 22.0 and 33.0 for comparison in ARDS; p = 0.022; median Ct values of 21.5 and 33.0 for comparison in acute kidney injury; p = 0.03; Fig 2B). Additionally, median initial Ct values were lower among participants falling into several adverse event categories compared to participants who did not fall into these categories, but these were not statistically significant differences (Fig 2B).

## Discussion

With COVID-19-specific antiviral therapy advancing in clinical development, the question of differentiating SARS-CoV-2 infection from that of influenza and assessing the clinical impact of the co-infection of both viruses will likely become highly relevant to care decisions worldwide [28]. We reported here the clinical characteristics and severity of 505 patients hospitalized with illness caused by H1N1, SARS-CoV-2, or co-infection of both viruses. Patients with H1N1 monoinfection exhibit more acute upper respiratory tract symptoms than that of SARS-CoV-2 monoinfection. In comparison with monoinfection, co-infection affected a predominantly older age group and was associated with poorer clinical outcome. Of note, we found that lower Ct values (higher viral loads in nasopharyngeal swabs of co-infected patients) may lead to higher number of adverse events and clinical symptoms. We identified acute kidney injury and ARDS defined by Berlin definition significantly associated with lower Ct values. Relationships between viral load and clinical outcomes have been described for the monoinfection of SARS-CoV-2 and other respiratory viruses [29], and our study shows that the same is true for co-infection of SARS-CoV-2 and H1N1 and further investigated the viral load trajectory.

Patients with COVID-19 often presented without fever [30]. In the current study, the absence of fever and upper respiratory tract signs and symptoms (eg, expectoration or sore throat) is more frequent in patients with SARS-CoV-2 infection than H1N1 patients, indicating that the target cells might be located in the lower airway [31]. Patients infected by SARS-CoV-2 had a higher probability of pneumonia than H1N1 patients, further confirming the difference in viral tropism between two viruses [30,32]. However, the pathogenic mechanism underlying these differences between the two viruses have not been fully determined and warrants further virological research. In this study, laboratory test findings of H1N1 group at admission tended to be more abnormal, especially concerning measurements of inflammatory markers (eg. C-reactive protein level and Procalcitonin level). These findings were in line with the study conducted by Li Y et al. [33]; and could lead to more common acute upper respiratory tract symptoms in hospitalized H1N1 patients [34]. A study conducted by Azevedo et al. further confirmed this phenomenon; in which tissue expression increasing of IL-8/IL-17A and a higher number of neutrophils were identified in samples from the H1N1 group compared to the COVID-19 group [35]. The diverse characteristics of infection provides the rationale for advancing differential treatment in efforts to improve outcomes of these two types of patients.

CRP and PCT, which are the severity indexes of pneumonia [36], were higher in co-infection patients, suggesting that co-infection of H1N1 and SARS-CoV-2 is more severe than monoinfection by SARS-CoV-2. A higher score on a seven-category ordinal scale was observed in patients with co-infection which indicated worse clinical status. Furthermore, after multi-factor analysis, co-infection was associated with higher odds of several adverse clinical outcomes. This finding was also in accordance with a previous report on Middle East Respiratory Syndrome Coronavirus [37]. The initial infection of H1N1, which may reduce the immune competence of the patients, can then aggravate experienced pneumonia by SARS-CoV-2 infection. Given the clinical severity of influenza co-infection in our study, increasing the rate of influenza screening in COVID-19 inpatients within a reasonable range to achieve early initiation of antiviral treatment for improvement of clinical outcome should be considered. The cost-effectiveness should also be taken into account when focusing on screening strategy. However, the similarity of clinical manifestations between H1N1 and SARS-CoV-2 makes the differentiation very difficult and there warrant research to understand if there is a profile of patients who would benefit more of test both viruses. Notably, co-infections more frequently occurred in patients with older age and more comorbidities possibly because of the relative lack of immunity to respirovirus in this population [38].

The American Thoracic Society (ATS) and Infectious Diseases Society of America (IDSA) recommended initial antibacterial treatment for influenza-positive adults with community-acquired pneumonia, because bacterial co-infections are a common and serious complication of influenza [39,40] and it is difficult to exclude the presence of bacterial co-infection in a patient with community-acquired pneumonia who tested positive for influenza virus [41]. In China, if co-bacterial infection cannot be ruled out in patients with COVID-19, empirical antibiotic, such as amoxicillin, azithromycin, or fluoroquinolones, was recommended for mild cases but broad-spectrum antibiotic covering all possible pathogens was suggested for severe cases [42]. In the present study, a higher rate of secondary bacterial infections was recorded among patients co-infected by SARS-CoV-2 and H1N1, suggesting that active antimicrobial stewardship in the co-infected cases should be recommend to ensure effective control of bacterial co-infection.

Few studies have examined possible relationships between clinical presentation and Ct value in patients co-infected by H1N1 and SARS-CoV-2. Among patients infected by single virus, one analysis found higher loads in patients with severe disease [29], while multiple studies in which these associations were examined found no significant relationship between viral load (or, separately, Ct value) and patient comorbidities [43], symptom status [44,45] and disease severity [46]. Our findings–that the initial Ct values are significantly lower among co-infected individuals suffering from adverse events and symptoms–represent a novel addition and enhanced granularity to this growing body of evidence. Our results bolster the clinical value of early access to SARS-CoV-2 nucleic acid detection testing, particularly for co-infected patients and reinforce existing guidance for testing. Further work will be required to directly link the nasopharyngeal Ct value presented here to tissue and peripheral response of severe COVID-19.

There are limitations in this study. First, it was a single-center analysis, and the features of the setting may not be representative of Chinese patients as a whole. As a retrospective study, the further clinical progression could not be collected. Second, we also failed to explore the relationships regarding 30-days mortality because of the limited number of death cases (n = 2). Third, our study only used A(H1N1)pdm09 but not influenza A(H3N2) or B viruses. Fourth, we haven’t performed any additional testing, e.g. ddPCR, to determine the viral loads in the samples which could enhance the accuracy of the comparison. Fifth, we didn’t include the information on influenza vaccination, which may affect the severity of the viral infection. Sixth, limited by the original data, no influenza case in this study was collected from December, which was against the epidemic rule of influenza. Furthermore, as a single-center study, the limited sample size may cause selection bias and insufficient estimation ability.

Our findings are relevant in the context of the COVID-19 pandemic. Co-infection affected a predominantly older age group and was associated with poorer clinical outcomes. The prevalence rate of co-infections in COVID-19 cases shows the importance of flu vaccination and warrants its increased coverage. Additionally, molecular diagnostic testing for H1N1 virus is recommended for COVID-19 inpatients to provide timely and appropriate treatments for improved outcomes. The Ct value collected with nasopharyngeal swabs in the early stage of co-infection could provide clues for the later trajectory of the disease.

## Supporting information

S1 Appendix The detailed protocol of the RT-PCR. (DOCX)
