# Supplementary material for: Clinical and virological impact of single and dual infections with influenza A (H1N1) and SARS-CoV-2 in adult inpatients
Source: PLoS Negl Trop Dis. 2021 Nov 29;15(11):e0009997. doi: 10.1371/journal.pntd.0009997 (PMC8659415; doi:10.1371/journal.pntd.0009997)
Supplement: S1 Appendix — (DOCX) [file pntd.0009997.s001.docx]

**S1 Appendix The detailed protocol of the RT-PCR**

The swabs were then placed into a collection tube with 150 μL of virus preservation solution, and total RNA was extracted by the respiratory sample RNA isolation kit (Zhongzhi, Wuhan, China) within 2 hours. Briefly, 40 μL of cell lysates were transferred into a collection tube followed by vortex for 10 seconds. After standing at room temperature for 10 minutes, the collection tube was centrifugated at 1000 rpm/min for 5 minutes. The suspension was used for real-time reverse transcription PCR assay of SARS-CoV-2 RNA. Two target genes, including open reading frame 1ab and nucleocapsid protein (N), were simultaneously amplified and tested during the real-time RT-PCR assay.
